# A case of *Ignatzschineria* bacteraemia in an unconscious man from the Netherlands

**DOI:** 10.1099/jmmcr.0.005043

**Published:** 2016-06-25

**Authors:** Edou Heddema, Frank Janssen, Harro van Westreenen

**Affiliations:** ^1^​ Department of Medical Microbiology and Infection Control, Zuyderland Medical Centre, Sittard-Geleen, the Netherlands; ^2^​ BaseClear B.V, Leiden, the Netherlands; ^3^​ Department of Intensive Care, Zuyderland Medical Centre, Sittard-Geleen, the Netherlands

**Keywords:** *Ignatzschineria*, maggots, bacteraemia, amoxicillin/clavulanic acid

## Abstract

**Introduction:**

*Ignatzschineria* species were previously known as *Schineria* species and are well known inhabitants of the larvae of the parasitic fly *Wohlfahrtia magnifica*.

**Case presentation:**

We report a case of *Ignatzschineria* species bacteraemia in a Dutch patient with a wound infested with maggots.

**Conclusion:**

In the past, these bacteria have been isolated from *Wohlfahrtia magnifica*, a fly not indigenous to The Netherlands. Other fly larvae such as the blowfly larvae probably infested the wound and harboured this *Ignatzschineria* strain which subsequently caused this bacteraemia. A two-week course of amoxicillin/clavulanic acid was given with good clinical response.

## Introduction

The bacterial genus *Ignatzschineria* was previously described by Toth *et al.* as *Schineria* but was renamed in 2007. The name *Ignatzschineria* was chosen to honour Ignatz Rudolph Schiner (1813–1873) who first described the fly *Wohlfahrtia magnifica* (Tóth *et al.*, 2007 ). *Ignatzschineria* species are well known inhabitants of the larvae of the parasitic fly *Wohlfahrtia magnifica* (the spotted flesh fly). Human infections with species of *Ignatzschineria* are rarely reported. In this article we describe a case of *Ignatzschineria* bacteraemia.

## Case report

A 71-year-old man was admitted to our emergency department after being found unconscious and hypoxemic in front of his house. He had a history of alcohol- and nicotine-abuse and lived a reclusive life. One week prior he had visited his general practitioner because of dyspnea. He was prescribed oral steroids and bronchodilators for presumed exacerbation of chronic obstructive pulmonary disease (COPD).

**Fig. 1.**
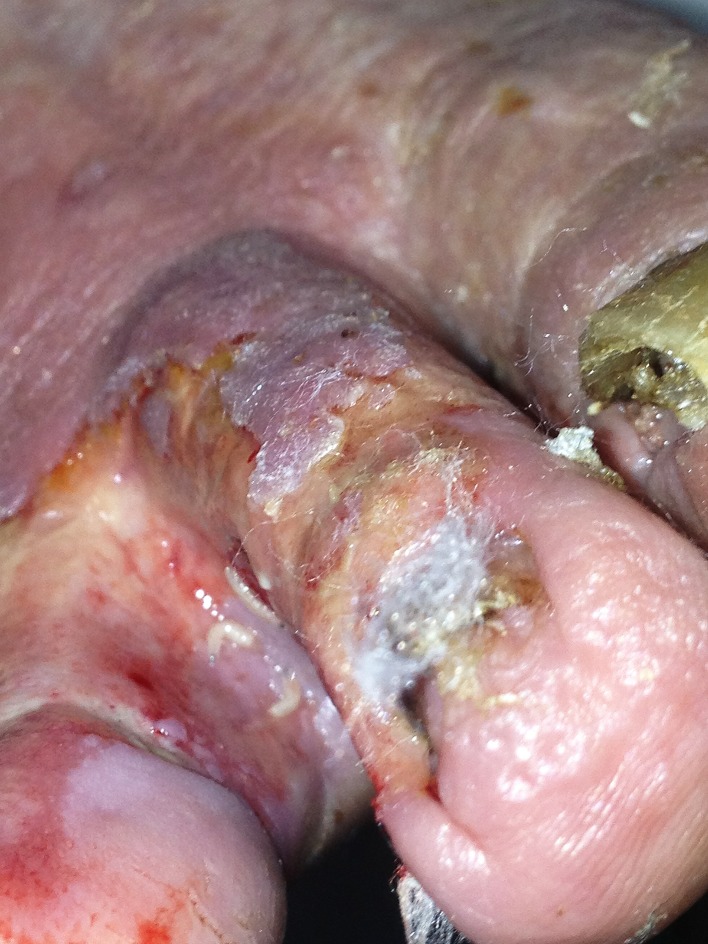
Maggot-infested wound (between toes).

**Fig. 2.**
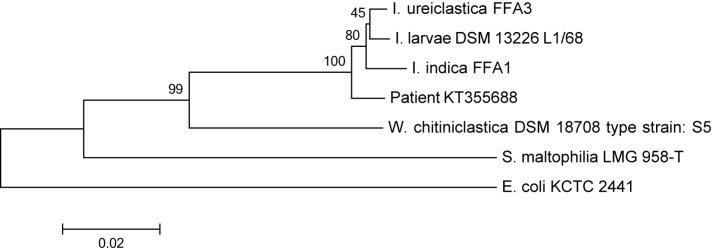
Phylogenetic tree displaying the full 16S rRNA gene sequences of the three species of the genus *Ignatzschineria*, our sample (GenBank accession no. KT355688) and some related species. The tree was created with mega 6.0 software using the neighbour-joining method, Jukes–Cantor model, and 1000 bootstraps. Bar, 0.02 substitutions per nucleotide.

On physical examination he looked ill, was haemodynamically stable and hypercapnic. His body temperature was 37.2 °C. His blood pressure was 154/66 mmHg, with a regular pulse of 116 beats min^−1^. He was tachypneic, respiration rate of 27 min^−1^, with a peripheral oxygen saturation of 97 % with a non-rebreathing mask. Lung auscultation revealed weak breath sounds. Further physical examination revealed a wound between his first and second toe on his right foot in which a great number of maggots were present (Fig. 1). Signs of chronic venous insufficiency were present, but no signs of arterial insufficiency. The remaining physical examination was unremarkable. Haematological and biochemical investigations showed a white blood cell count of 17.9×10^9^ l^−1^[reference range (ref.) 4–10×10^9^ l^−1^], with 81 % neutrophils (ref. 40–75 %), C-reactive protein 51 mg l^−1^ (ref. <10), glucose level of 257.6 mg dl^−1^ (ref. 72.1–115.1) and otherwise normal results for haemoglobin level, platelet count, liver enzymes, electrolytes and creatinine. Arterial blood-gas analysis showed a marked respiratory acidosis with a pH of 7.19 and pCO2 of 12.1 kPa. A chest X-ray did not reveal signs of pneumonia. He was admitted to our Intensive Care Unit and non-invasive ventilation was started with a diagnosis of exacerbation of his COPD without an apparent trigger, possibly upper respiratory tract infection. Steroids, bronchodilators and a broad-spectrum antibiotic (amoxicillin/clavulanic acid) were given. Two days later he was transferred to the pulmonology ward. Two blood culture sets each containing one aerobic and one anaerobic bottle (BACTEC Plus Aerobic/F and Anaerobic/F Culture Vials using the BACTEC FX system; Becton Dickinson) drawn upon admission revealed growth of Gram-negative rods in one of the aerobic bottles after incubation for 48 h. Upon subculturing of the Gram-negative rod, an oxidase-positive, aerobic, catalase-positive strain was found. Determination by Vitek2Combo with ID-GN card (bioMérieux) revealed *Acinetobacter iwoffii* with 98 % probabil ity. API NE tests (bioMérieux) could not assign the strain to a specific species. The above results were conflicting with colony morphology and oxidase reaction. A RapID-ANA test (Remel) was done to test for enzymatic reactions that could possibly provide a clue to the identification. Positive reactions for urease, arginine and serine hydrolysis were found. Although we even considered this strain to be a non-fermentative contaminant, the combination of the positive oxidase reaction and the growth characteristics finally let us consider *Wohlfahrtiimonas* species as we knew the patient had a wound full with maggots. However , a positive urease reaction is not compatible with *Wohlfahrtiimonas*. We offered the strain to an external laboratory (BaseClear, Leiden, The Netherlands) for identification by matrix-assisted laser desorption/ionization time-of-flight mass spectrometry (MALDI-TOF MS) (VITEK MS; bioMérieux) and MicroSEQ analysis, a 16S rRNA gene amplification and sequencing method (Life Technologies). MALDI-TOF MS did not reveal a reliable result (VITEK MS industry database v2.0) and even the MicroSEQ analysis with the validated database was unsuccessful. To our complete surprise a species of the genus*Ignatzschineria* was identified when the 489 basepair 16S rRNA gene sequence was blasted against the NCBI and SMARTgene databases. The colony morphology, growth characteristics, oxidase and urease reaction were all compatible with this identification as well as the clinical story. The complete 16S rRNA gene was amplified and analysed. The resulting sequence (1392 nucleotides) showed 98.7, 98.5 and 98.2 % similarity, respectively to the type strains of *Ignatzschineria ureiclastica*, *Ignatzschineria larvae* and *Ignatzschineria indica* (GenBank accession numbers EU008089, AJ252143 and EU008088). Fig. 2 shows a phylogenetic tree displaying the relations with close proximate species. The sequence was submitted to the GenBank database and assigned accession number KT355688. Antimicrobial susceptibility testing performed by Etest (bioMérieux) revealed minimal inhibitory concentrations (MIC) for amoxicillin/clavulanic acid of 0.25 mg l^−1^, amoxicillin 0.047 mg l^−1^ and ciprofloxacin 0.064 mg l^−1^. A beta-lactamase test (Oxoid) was positive. According to EUCAST non-species related breakpoints all these antibiotics could be considered sensitive based on Pk/Pd data. The positive beta-lactamase test argues against amoxicillin as appropriate treatment. The *in vitro* sensitivity to amoxicillin by Etest in conjunction with a positive beta-lactamase test is suggestive of an inducible beta-lactamase. A culture of the wound on the right foot of the patient taken two days after start of antimicrobial treatment and admission did only reveal *Providencia stuartii*. Even with the knowledge of the blood culture result we could not find species of the genus *Ignatzschineria* in this culture. A two-week course of amoxicillin/clavulanic acid was completed with good clinical response. The wound on the patient's foot was successfully treated with application of povidone-iodine (betadine) and low adherent highly absorbent (melonin) dressings. His pulmonary condition improved, elevated glucose level and venous insufficiency were taken care of and he gradually improved and was discharged three weeks after admission.

## Discussion

To our knowledge, this is the first reported human case of *Ignatzschineria* bacteraemia from the Netherlands. In 2007, Toth *et al.* described four *Ignatzschineria* strains isolated from fly larvae. The type species was named *Ignatzschineria larvae*. In 2011, two additional species were added: *I. indica* and *I. ureiclastica* (Gupta, *et al.*, 2011). Members of the genus *Ignatzschineria* are aerobic, catalase- and oxidase-positive. Strains are mainly assacharolytic and difficult to identify by standard medical microbiology identification methods. MALDI-TOF MS analysis was unsuccessful too. Currently 16S rRNA gene sequencing seems the most accurate method for clinical diagnostic laboratories. Taxonomically this genus is closely related to the genus *Wohlfahrtiimonas*, also a very recently recognized genus associated with maggots (TothTóth, *et al.*, 2008; Rebaudet, *et al.*, 2009). Only four other case reports describe *Ignatzschineria* spp. bacteraemia in five different patients. These reports also mentioned neglected wounds with maggots as probable source of infection (Barker, *et al.*, 2014 ; Le Brun, *et al.*, 2015; Roudiere, *et al.*, 2007 ;(Maurin, *et al.*, 2007). We did not analyse or identify the maggots in the wounds as they were thrown away after wound cleaning. Therefore we cannot prove that the larvae were definitely from the fly *Wohlfahrtia magnifica*. In the Netherlands *Wohlfahrtia magnifica* is not an indigenous species. Probably the maggots were derived from other flies such as the green blowfly (*Lucilia sericata).* Species of the genus *Ignatzschineria* and also the species *Providencia stuartii* – as cultured from our patient's wound – are found in relatively high abundances in the salivary gland of *L. sericata* (Singh, *et al.*, 2015). Barker, *et al.* (2014) described a case of *I. indica* associated with blowfly larva. However, our strain was genetically most related to *I. ureiclastica*, but the antimicrobial susceptibility for amoxicillin/clavulanic acid and ciprofloxacin is in concordance with the description of *I. larvae* (Gupta, *et al.*, 2011) . It should be mentioned that two of the four case reports describing *Ignatzschineria* infections in humans were written before the description of *I. indica* and *I. ureiclastica*. Probably, more extensive typing tools like whole-genome sequencing are needed to exactly elucidate the taxonomic status of these strains and to link them to the most preferred host fly larvae. The wound culture was negative for species of the genus *Ignatzschineria* probably because it was taken three days after admission and start of antibiotic therapy. In our patient a good clinical response was seen on amoxicillin/clavulanic acid therapy. We were very cautious to rely completely on the *in vitro* antimicrobial susceptibility tests as they are only based on Pk/Pd assumptions. The wound culture grew *Providencia stuartii*. This amoxicillin/clavulanic acid-resistant bacterium was treated locally with wound dressings and good clinical response. In conclusion, Gram-negative rods in (blood) cultures from patients with myasis could be species of the genus *Ignatzschineria*. Only three well established species of this genus have been described. They are difficult to identify as they are not incorporated in the respective databases for standard biochemical identification tests and MALDI-TOF MS.
